# Multiscale Prediction of Equivalent Elastic Properties of Carbon/Glass Hybrid Composite Laminates

**DOI:** 10.3390/polym18151882

**Published:** 2026-07-31

**Authors:** Qi Luo, Lexian Zhang, Fengjia Zhang, Tianke Tuo, Yibo Wu, Anxin Ding

**Affiliations:** 1 Luoyang Ship Material Research Institute, Luoyang 471023, China; lqymydtt@163.com (Q.L.); fengjiazhang2024@163.com (F.Z.); ttk747101060@163.com (T.T.); 15803797972@163.com (Y.W.); 2 School of Materials Science and Engineering, Wuhan University of Technology, Wuhan 430070, China; 3 Hubei Longzhong Laboratory, Wuhan University of Technology Xiangyang Demonstration Zone, Xiangyang 441100, China

**Keywords:** representative volume element (RVE), numerical simulation, hybrid composites, multi-scale homogenization

## Abstract

A multi-scale framework is proposed for predicting the equivalent engineering elastic constants of carbon/glass hybrid composite laminates with different fiber volume fractions *V_f_* by integrating multiscale finite element homogenization with analytical micromechanics. Biaxial carbon fiber fabric reinforced vinyl ester composites are used as the target material system, and representative volume element (RVE) models are constructed sequentially at the lamina, laminate, and hybrid-laminate scales. At the lamina scale, the analytical self-consistent field micromechanics (SCFM) approach and the RVE method give highly consistent predictions for the fiber-dominated longitudinal modulus *E*_1_ and in-plane shear modulus *G*_12_, with deviations below 2%. However, the SCFM approach overestimates the transverse modulus *E*_2_ and transverse Poisson’s ratio *ν*_23_ as the *V_f_* rises. The unidirectional lamina constants predicted by SCFM and RVE are then used as inputs for an analytical solution for predicting elastic constants (ASPE) and a finite-element-analysis-based micromechanics (FEAM) model of laminate and hybrid-laminate. At the laminate scale, the moduli predicted by ASPE(SCFM) and ASPE(RVE) differ by less than 2%, whereas the out-of-plane properties are sensitive to the transverse lamina inputs. Experimental validation shows that the *E_x_* values predicted by ASPE(RVE) and FEAM(RVE) deviate from the measured average by approximately 3.5%, with good agreement also obtained for *G_xy_* and *ν_xy_*. At the hybrid-laminate scale, ASPE(RVE) and FEAM(RVE) exhibit only minor differences in estimating the in-plane moduli, out-of-plane moduli, and Poisson’s ratios.

## 1. Introduction

Accurate prediction of effective mechanical properties is essential for the preliminary design of composite structures [1,2]. For multi-ply laminates, conventional ply-by-ply modelling is computationally demanding, especially during early-stage structural optimization [3,4]. Therefore, heterogeneous laminates are frequently treated as equivalent homogeneous anisotropic media to reduce computational effort. The accuracy of the resulting engineering elastic constants directly influences the reliability of predicted load-transfer efficiency, stress distribution, and deformation compatibility. Early analytical formulations offer useful approximations of elastic moduli and hygrothermal properties [5,6]; however, they were constrained by idealized periodic assumptions and simple geometric configurations. As mesostructures become more complex, multiscale micromechanics based on RVEs has emerged as an efficient tool for establishing quantitative links between microscopic constituents, including fibers, matrices, and interfaces, and macroscopic equivalent responses. Combining FE analysis with micromechanics has become a standard approach for predicting the homogenized properties of plies and laminates. In this approach, an RVE is constructed and appropriate boundary conditions are imposed to resolve the micromechanical fields, from which the macroscopic constitutive relationship and engineering constants are derived. Adaptive finite element techniques can further enhance numerical accuracy [7]. Sun et al. [8] verify the accuracy of this approach through both theoretical and experimental analyses. Garnich et al. extract single-wavelength fiber-waviness RVEs and evaluate the effects of waviness ratio and fiber volume fraction on macroscopic stiffness degradation. For fabric-reinforced composites, two- and three-dimensional finite element models have been used to predict the elastic properties of plain-weave and three-dimensional braided structures [3,9]. Furthermore, Lu et al. [10] incorporate interface elements to account for the contribution of the fiber–resin interface, whereas Ghosh et al. [11] propose a modulus prediction method based on polygonal unit cells. Trias et al. [12] demonstrate that periodic models can accurately represent the stress–strain response of unidirectional plies while maintaining high computational efficiency. The effects of fiber arrangement, fiber shape, and fiber volume fraction on elastic moduli have also been examined widely [2,13].

Current studies on stiffness estimation have mainly focused on single-ply or unit-cell scales. In contrast, cross-scale prediction of the overall engineering constants of hybrid laminates, particularly thick-walled structures, remains insufficient. In the preliminary design of large composite structures, equivalent laminate-level parameters are typically required rather than individual ply properties. Sun and Li [14], and Sun and Liao [15] proposed early approaches for evaluating three-dimensional effective elastic constants of laminates. More recently, substantial progress has been made in multiscale determination of laminate-level equivalent properties. Shu and Stanciulescu [13] establish a multiscale homogenization method for fiber-reinforced composites, offering a theoretical framework for progressive modulus prediction from laminae to laminates. Ge et al. [16] introduce void defects into a three-dimensional five-directional braided RVE and quantify their influence on macroscopic elastic constants. Praud et al. [17] simulate the damage and rate-dependent behavior of thermoplastic woven laminates using a fully coupled multiscale model. Wang et al. [18] construct a novel three-dimensional RVE to evaluate micro-damage initiation in Z-pin-reinforced laminates. Heide-Jørgensen et al. [9] present a three-dimensional multiscale homogenization model to assess stiffness reduction caused by fiber–matrix debonding in hybrid woven composites. To improve computational efficiency, Mathew et al. [19] propose an adaptive-importance-sampling neural-network framework for reliability prediction of variable-stiffness laminates, and Logarzo et al. [20] construct machine-learning-based constitutive laws for inelastic homogenization. Agarwal et al. [21] review advances from classical micromechanics to artificial-intelligence-assisted multiscale models, emphasizing the importance of method integration and computational efficiency for engineering applications. Overall, multiscale equivalent-property estimation has gradually expanded from elastic constants to coupled evaluations of thermoelastic parameters and strength-related responses [22,23,24,25]. Wisnom et al. [26] reveal the significant influence of component thickness on cure-induced spring-in through shear-lag analysis, indicating that the mechanical response of thick-walled composite structures cannot be reduced to a simple linear superposition of single-ply behavior. Therefore, a cross-scale prediction method that bridges the lamina and laminate levels is needed for accurate equivalent-property evaluation of hybrid thick laminates.

This study develops a multi-scale method for predicting the equivalent engineering elastic constants of hybrid composites based on multiscale homogenization. Carbon fiber reinforced vinyl ester hybrid composites are chosen as the target material system. FE RVE models and analytical models are established for unidirectional laminae of BCFRP, laminates of BCFRP, and carbon/glass hybrid laminates. This framework enables progressive prediction of equivalent moduli from fiber/matrix constituents to hybrid composite structures. Numerical homogenization and theoretical predictions are compared at each scale and further validated against experimental results. The proposed method provides methodological support for accurate and efficient structural optimization of hybrid composite laminates.

## 2. Materials and Methods

### 2.1. Materials

Vinyl ester resin serves as the matrix, and biaxial T700-grade carbon fiber fabrics and S-grade high-strength glass fiber fabric are used as the reinforcement. Akzo Nobel Butanox M50 and cobalt octoate act as the curing agent and accelerator, respectively. The material properties of the constituents are listed in Table 1.

### 2.2. Specimen Preparation

**Figure 1:**
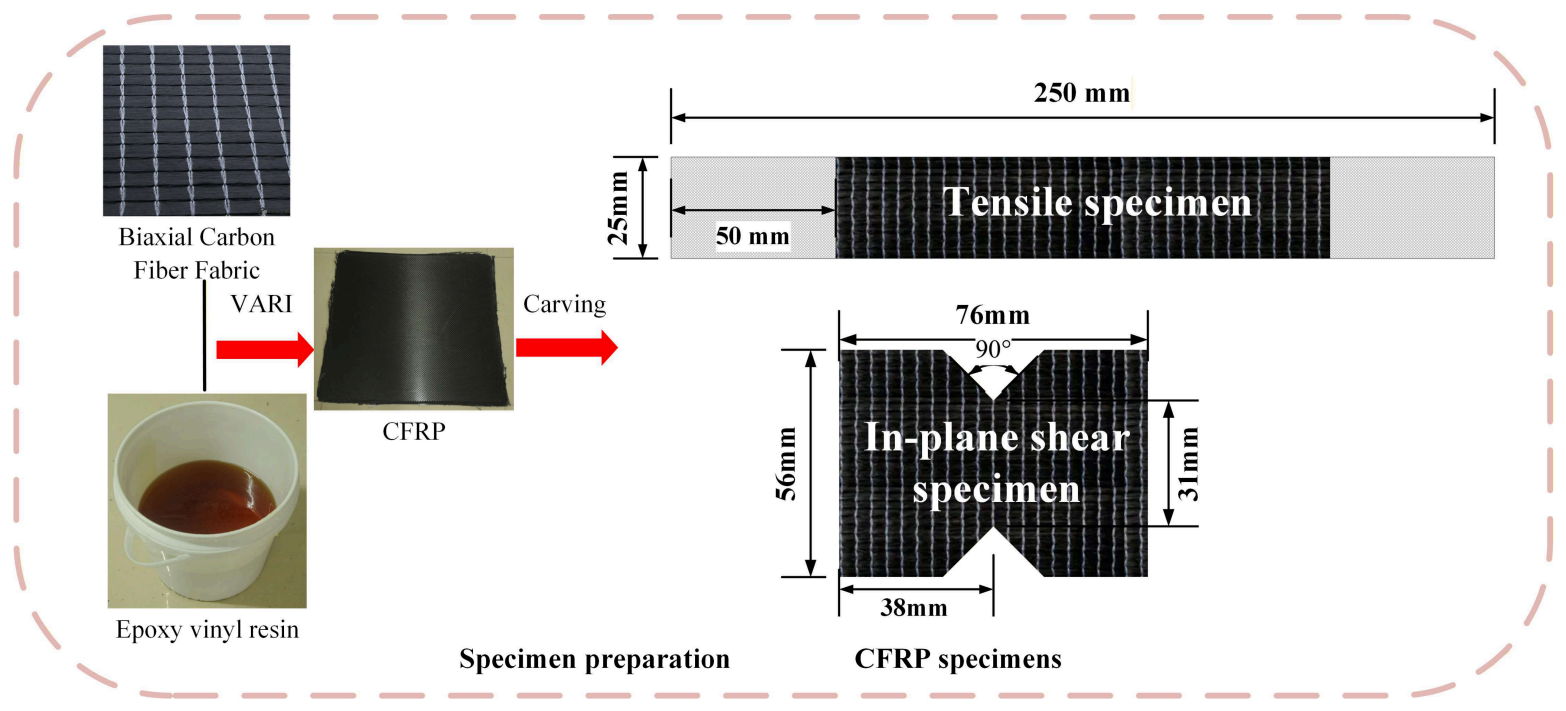
Manufacturing process and geometries of the BCFRP tensile and in-plane shear specimens.

The resin, curing agent, and accelerator were mixed at a mass ratio of 100:2:0.2 and degassed in a vacuum oven for 10 min. BCFRP laminates with thicknesses of 2 and 4 mm were fabricated using the vacuum-assisted resin infusion process. After demolding, the laminates were dried at 50 °C for 48 h to remove surface moisture. Standard specimens were machined with an engraving machine. Figure 1 displays their manufacturing process and dimensions. The resulting fiber mass fraction of the BCFRP laminates was 62.1%, equivalent to a fiber volume fraction of *V_f_* = 50.8% based on Table 1. The thickness of a single BCFRP layer [0°/90°] was 0.425 mm.

### 2.3. Mechanical Testing

A LE055 universal testing machine with a 100 kN load capacity (Lishi Scientific Instruments Co., Ltd., Shanghai, China) was employed to measure the tensile and in-plane shear properties of the BCFRP specimens. Seven specimens were tested for each group. Specimens showing unacceptable failure modes were excluded, and at least five valid results were kept for each group. All tests were conducted at a crosshead speed of 2 mm/min. Tensile tests were conducted according to ASTM D3039 [27], and in-plane shear tests followed ASTM D7078 [28]. ASTM D3039 and ASTM D7078 correspond with the standard test methods for tensile properties and V-notched rail shear properties of polymer matrix composite materials, respectively.

## 3. Models and Theory

### 3.1. Procedure for Predicting Equivalent Engineering Elastic Constants of Hybrid Laminates

**Figure 2:**
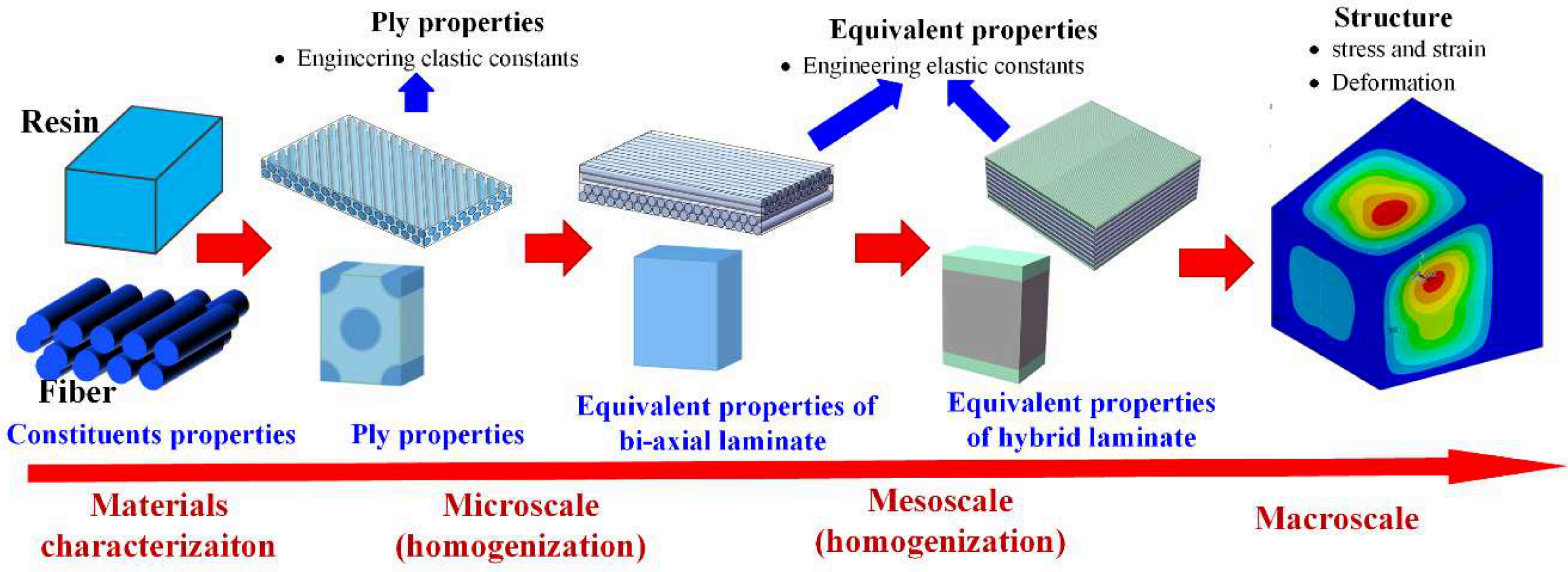
Technical route for predicting equivalent engineering elastic constants of hybrid composites.

Based on the constituent material properties, the stiffness of a unidirectional BCFRP lamina was first determined using the RVE model and micromechanics-based analytical models. Subsequently, following the layup configuration, a BCFRP laminate RVE and the corresponding analytical model were established to evaluate the equivalent engineering elastic constants at the laminate scale. Finally, the layup sequence and structural dimensions of the hybrid composite were used as input, together with the predicted BCFRP core properties and the Glass Fiber Reinforced Polymer composite (GFRP) skin properties, to evaluate the equivalent engineering constants of the hybrid laminate. The overall technical route is shown in Figure 2.

### 3.2. RVE Models

#### 3.2.1. RVE Model of the Unidirectional Lamina

**Figure 3:**
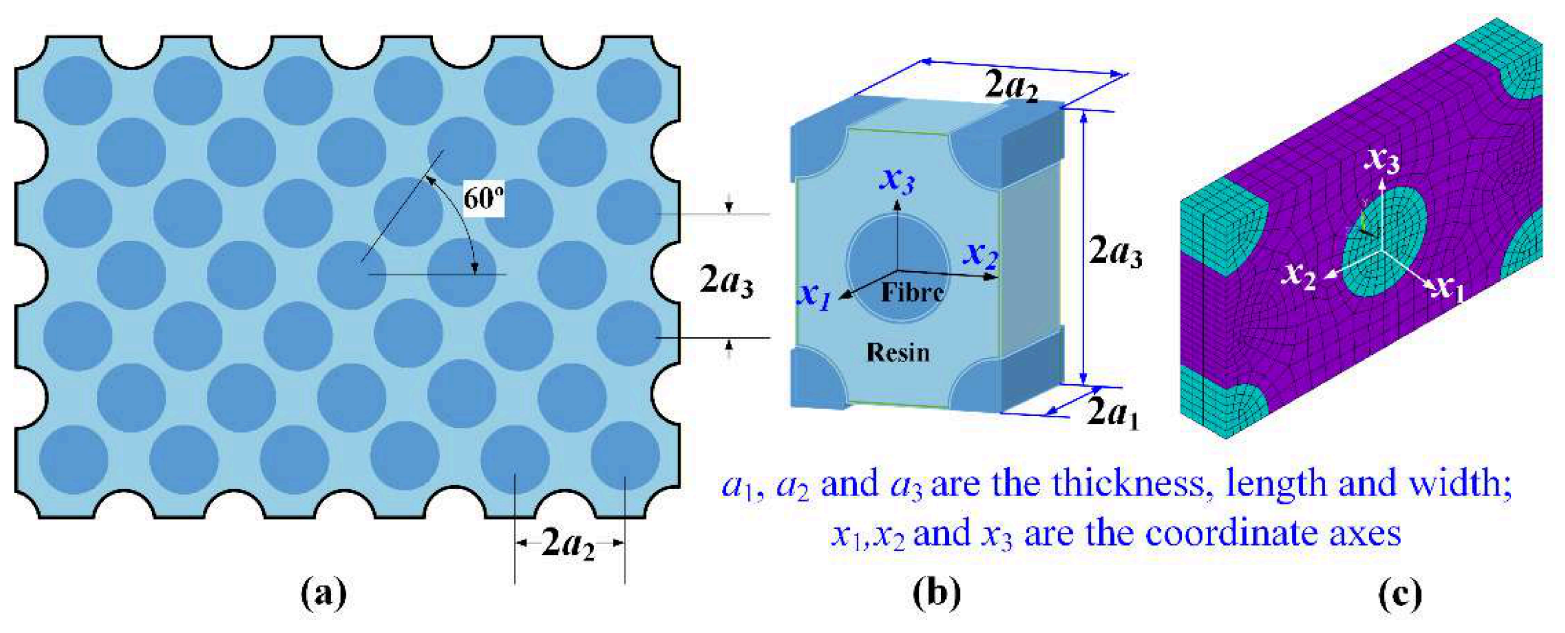
RVE model of a unidirectional lamina: (**a**) hexagonal fiber arrangement, (**b**) geometric RVE of the lamina, and (**c**) FE model of the RVE.

The RVE model of a unidirectional BCFRP lamina was built to represent the mesoscopic fiber–matrix architecture. Because the fibers were treated as regularly and uniformly distributed in the matrix, the lamina was considered as a periodic assembly of RVEs. A hexagonal fiber-packing configuration was adopted for the unidirectional lamina, as shown in Figure 3a, and the corresponding RVE is illustrated in Figure 3b. The carbon fiber filament diameter was taken as *d_c_*_f_ = 7 μm. The RVE volume was defined as *V* = 2*a*_1_*a*_2_2*a*_3_, and the *V_f_* was derived from the geometric parameters. For the manufactured BCFRP specimen, *V_f_* was 50.8%. The values of *a*_2_ and *a*_3_ were obtained according to the prescribed fiber volume fraction. Because the fiber length has a negligible influence on the predicted elastic moduli, *a*_1_ can be selected arbitrarily; in this study, *a*_1_ = *a*_2_/4 was adopted in the simulations. The resulting FE RVE model is displayed in Figure 3c, and C3D8 elements were implemented in ABAQUS 2019. Linear hexahedral C3D8 elements were selected because they provide sufficient accuracy for linear elastic problems and naturally accommodate the periodic boundary conditions applied to the RVE faces. Both the fiber and matrix were modeled as homogeneous, linear elastic materials, with perfect interfacial bonding and no voids or defects. The relationship between the macroscopic stress–strain response of the lamina and the constituent properties is given by Equation (1).

(1) $$\begin{array}{c} {\bar{\sigma }}_{ij}=\frac{1}{V}\int_{V}\sigma_{ij}\left(x_{1},x_{2},x_{3}\right)dV=\frac{1}{V}\sum V_{\alpha }\sigma_{ij}^{\alpha }\\ {\bar{\varepsilon }}_{ij}=\frac{1}{V}\int_{V}\varepsilon_{ij}\left(x_{1},x_{2},x_{3}\right)dV=\frac{1}{V}\sum V_{\alpha }\varepsilon_{ij}^{\alpha } \end{array}$$

where *σ* and *ε* represent stress and strain, respectively; subscripts *i* and *j* correspond to coordinate axes (*x*_1_, *x*_2_, *x*_3_) directions; *V* is the total volume of the RVE; and *V_α_* is the volume of each constituent material. The constituent materials of the unidirectional lamina considered here exhibit transversely isotropic behavior, and their constitutive relations can be written as Equation (2).

(2) $$\left\{\begin{array}{c} {\bar{\sigma }}_{1}\\ {\bar{\sigma }}_{2}\\ {\bar{\sigma }}_{3}\\ {\bar{\sigma }}_{23}\\ {\bar{\sigma }}_{13}\\ {\bar{\sigma }}_{12} \end{array}\right\}=\left[\begin{array}{cccccc} C_{11} & C_{12} & C_{12} & 0 & 0 & 0\\ C_{12} & C_{22} & C_{23} & 0 & 0 & 0\\ C_{12} & C_{23} & C_{22} & 0 & 0 & 0\\ 0 & 0 & 0 & \frac{1}{2}\left(C_{22}-C_{23}\right) & 0 & 0\\ 0 & 0 & 0 & 0 & C_{66} & 0\\ 0 & 0 & 0 & 0 & 0 & C_{66} \end{array}\right]\left\{\begin{array}{c} {\bar{\varepsilon }}_{1}\\ {\bar{\varepsilon }}_{2}\\ {\bar{\varepsilon }}_{3}\\ {\bar{\varepsilon }}_{23}\\ {\bar{\varepsilon }}_{13}\\ {\bar{\varepsilon }}_{12} \end{array}\right\}$$

In Equation (2), **[C]** denotes the macroscopic stiffness matrix of the lamina, and the five independent stiffness coefficients are derived from the microscale constituent properties. Axes 1, 2, and 3 define the principal material coordinate system of the lamina, corresponding to the longitudinal, transverse, and through-thickness directions, respectively, as shown in Figure 3c. The RVE is subjected to strain-controlled boundary conditions, in which each macroscopic strain component was prescribed sequentially as a unit value, as expressed in Equation (3).

(3) $$\begin{array}{c} u_{i}\left(a_{1},x_{2},x_{3}\right)-u_{i}\left(-a_{1},x_{2},x_{3}\right)=2a_{1}\varepsilon_{i1}^{0}\left(-a_{2}\leq x_{2}\leq a_{2}-a_{3}\leq x_{3}\leq a_{3}\right)\\ u_{i}\left(x_{1},a_{2},x_{3}\right)-u_{i}\left(x_{1},-a_{2},x_{3}\right)=2a_{2}\varepsilon_{i2}^{0}\left(-a_{1}\leq x_{1}\leq a_{1}-a_{3}\leq x_{3}\leq a_{3}\right)\\ u_{i}\left(x_{1},x_{2},a_{3}\right)-u_{i}\left(x_{1},x_{2},-a_{3}\right)=2a_{3}\varepsilon_{i3}^{0}\left(-a_{1}\leq x_{1}\leq a_{1}-a_{2}\leq x_{2}\leq a_{2}\right) \end{array}$$

On the three symmetry planes of the RVE, namely *x*_1_ = 0, *x*_2_ = 0 and *x*_3_ = 0, the corresponding normal displacement components are fixed. Specifically, *u*_1_ = 0 is imposed on *x*_1_ = 0, *u*_2_ = 0 on *x*_2_ = 0, and *u*_3_ = 0 on *x*_3_ = 0. Coupling constraints are further imposed on the three pairs of parallel faces to ensure compatible nodal displacements. When one strain component is prescribed, and the other five strain components are set to zero, the strain expression in Equation (1) simplifies to Equation (4).

(4) $${\bar{\varepsilon }}_{11}=\frac{1}{V}\int_{V}\varepsilon_{11}\left(x_{1},x_{2},x_{3}\right)dV=\varepsilon_{11}^{0}$$

Equation (2) can also be rewritten as Equation (5).

(5) $$C_{i1}={\bar{\sigma }}_{i}=\frac{1}{V}\int_{V}\sigma_{i}\left(x_{1},x_{2},x_{3}\right)dV$$

By applying the remaining five strain states in the same manner and calculating the volume-averaged stress under each boundary condition, the other stiffness coefficients of the macroscopic stiffness matrix are obtained. The macroscopic moduli, namely the engineering elastic constants of the unidirectional lamina, are subsequently retrieved from the relationship between the compliance matrix and the engineering constants. For the hexagonal-packing RVE model, the equivalent fiber diameter *d_f_* is calculated using Equation (6). For the hexagonal packing RVE, with the length and width taken as $\sqrt{3}$ and 1, respectively, the equivalent fiber diameter *d_f_* is calculated using Equation (6). Note that *d_f_* is expressed in the same units as the RVE length and width.

(6) $$d_{f}=\sqrt{\frac{2\sqrt{3}V_{f}}{\pi }}$$

Finally, the engineering elastic constants of the unidirectional lamina are calculated using Equation (7).

(7) $$\begin{array}{ll} E_{1}=C_{11}-\frac{{2C}_{11}^{2}}{C_{22}+C_{23}}; & E_{2}=\frac{\left[C_{11}\left(C_{22}+C_{23}\right)-{2C}_{12}^{2}\right]\left(C_{22}+C_{23}\right)}{\left(C_{11}C_{22}+{2C}_{12}^{2}\right)}=E_{3}\\ \nu_{12}=\frac{C_{12}}{C_{22}+C_{23}}=\nu_{13}; & \nu_{23}=\frac{\left(C_{11}C_{23}-{2C}_{12}^{2}\right)}{\left(C_{11}C_{22}+{2C}_{12}^{2}\right)}\\ G_{12}=C_{66}=G_{13}; & G_{23}=\frac{E_{3}}{2(1+\nu_{23})} \end{array}$$

#### 3.2.2. RVE Models of the Laminate and Hybrid Laminates

**Figure 4:**
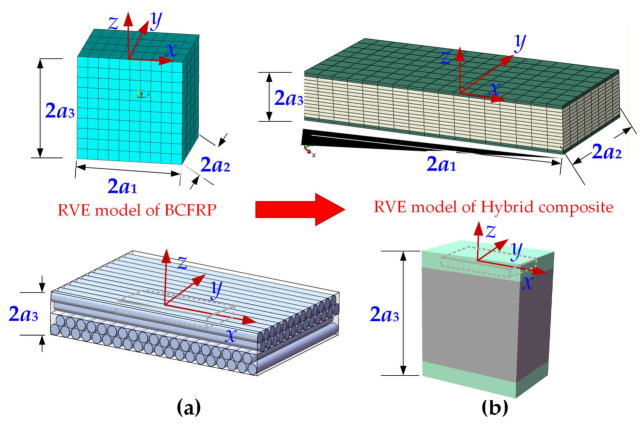
RVE models of the laminate and hybrid laminate: (**a**) BCFRP laminate and (**b**) hybrid laminate.

The equivalent engineering elastic constants of a laminate depend on both the lamina properties and the layup configuration. An accurate laminate-level RVE should link the response of each lamina to the overall macroscopic behavior of the laminate. The RVE models developed for the BCFRP laminate and hybrid laminate are shown in Figure 4. In these models, the thickness direction coincides with the laminate thickness direction, whereas the in-plane length and width can be selected according to the homogenization requirement. To reduce edge effects, both the in-plane length and width are set to five times the laminate thickness *t*. Within the laminate and hybrid-laminate RVEs, the displacement and stress fields are taken as continuous, and the macroscopic stress–strain relationship is obtained by volume averaging the elemental stresses and strains, as given in Equation (8). The constitutive relationship of the laminate and hybrid laminate is defined as Equation (9).

(8) $$\begin{array}{c} {\bar{\sigma }}_{ij}=C_{ij}=\frac{1}{V}\int_{V}\sigma_{\alpha }\left(x,y,z\right)dV \end{array}$$

(9) $$\left\{\begin{array}{c} {\bar{\sigma }}_{x}\\ {\bar{\sigma }}_{y}\\ {\bar{\sigma }}_{z}\\ {\bar{\sigma }}_{yz}\\ {\bar{\sigma }}_{xz}\\ {\bar{\sigma }}_{xy} \end{array}\right\}=\left[\begin{array}{cccccc} C_{xx} & C_{xy} & C_{xz} & 0 & 0 & 0\\ C_{xy} & C_{yy} & C_{yz} & 0 & 0 & 0\\ C_{xz} & C_{yz} & C_{zz} & 0 & 0 & 0\\ 0 & 0 & 0 & C_{yz} & 0 & 0\\ 0 & 0 & 0 & 0 & C_{xz} & 0\\ 0 & 0 & 0 & 0 & 0 & C_{xy} \end{array}\right]\left\{\begin{array}{c} {\bar{\varepsilon }}_{x}\\ {\bar{\varepsilon }}_{y}\\ {\bar{\varepsilon }}_{z}\\ {\bar{\varepsilon }}_{yz}\\ {\bar{\varepsilon }}_{xz}\\ {\bar{\varepsilon }}_{xy} \end{array}\right\}$$

To obtain the macroscopic stiffness matrix **[*C*]** of the laminate and hybrid laminate, the same strain-controlled strategy as that employed for the unidirectional lamina RVE is adopted. Unit macroscopic strain components are applied sequentially to the RVE, with one strain component prescribed at a time and all other components set to zero. For each loading case, the volume-averaged stress is computed, and the corresponding column of **[*C*]** is evaluated. The equivalent engineering elastic constants of the macroscopic RVE are then obtained from Equation (7). This FE-based approach for predicting equivalent engineering elastic constants of laminate and hybrid-laminate RVEs is referred to as the FEAM. The thicknesses of a layer of BCFRP and a GFRP are 0.425 and 0.2 mm, respectively. For the BCFRP laminate, a single-layer biaxial RVE is constructed directly, as illustrated in Figure 4a. The hybrid composite has a layup sequence of two satin-weave GFRP layers, twelve BCFRP layers, and two satin-weave GFRP layers, with a total thickness of 5.9 mm. Accordingly, the hybrid-composite RVE is simplified as a three-layer GFRP–CFRP–GFRP configuration, as depicted in Figure 4b, with each layer thickness determined from the actual stacking proportion. All FE simulations are performed in ABAQUS using C3D8 elements. In the fabricated hybrid structure, the fiber volume fractions of the surface GFRP layer and the BCFRP core are 43.0% and 50.8%, respectively.

### 3.3. Analytical Models

#### 3.3.1. Analytical Prediction of Lamina Engineering Elastic Constants

In this study, the SCFM model is used to evaluate the engineering elastic constants of the lamina. The corresponding expressions are given in Equation (10) [29].

(10) $$\begin{array}{c} \begin{array}{c} E_{1}=E_{1f}V_{f}+E_{r}\left(1-V_{f}\right)+\left[\frac{4\left(\nu_{r}-{\nu^{2}}_{12f}\right)k_{f}k_{r}G_{r}\left(1-V_{f}\right)V_{f}}{\left(k_{f}+G_{r}\right)k_{r}+\left(k_{f}-k_{r}\right)G_{r}V_{f}}\right]\\ E_{2}=E_{3}=\frac{1}{\left(4k_{T}{)}^{-1}+(4G_{23}{)}^{-1}+(\nu_{12}^{2}/E_{11}\right)}\\ G_{12}=G_{13}=G_{r}\left[\frac{\left(G_{12f}+G_{r}\right)+\left(G_{12f}-G_{r}\right)V_{f}}{\left(G_{12f}+G_{r}\right)-\left(G_{12f}-G_{r}\right)V_{f}}\right]\\ G_{23}=\frac{G_{r}\left[k_{r}\left(G_{r}+G_{23f}\right)+2G_{23f}G_{r}+k_{r}\left(G_{23f}-G_{r}\right)V_{f}\right]}{k_{r}\left(G_{r}+G_{23f}\right)+2G_{23f}G_{r}-\left(k_{r}+2G_{r}\right)\left(G_{23f}-G_{r}\right)V_{f}}\\ E_{2}=E_{3}=\frac{1}{\left(4k_{T}{)}^{-1}+(4G_{23}{)}^{-1}+(\nu_{12}^{2}/E_{11}\right)}\\ G_{12}=G_{13}=G_{r}\left[\frac{\left(G_{12f}+G_{r}\right)+\left(G_{12f}-G_{r}\right)V_{f}}{\left(G_{12f}+G_{r}\right)-\left(G_{12f}-G_{r}\right)V_{f}}\right]\\ G_{23}=\frac{G_{r}\left[k_{r}\left(G_{r}+G_{23f}\right)+2G_{23f}G_{r}+k_{r}\left(G_{23f}-G_{r}\right)V_{f}\right]}{k_{r}\left(G_{r}+G_{23f}\right)+2G_{23f}G_{r}-\left(k_{r}+2G_{r}\right)\left(G_{23f}-G_{r}\right)V_{f}}\\ \nu_{12}=\nu_{13}=\nu_{12f}V_{f}+\nu_{r}\left(1-V_{f}\right)+\left[\frac{\left(\nu_{r}-\nu_{12f}\right)\left(k_{r}-k_{f}\right)G_{r}\left(1-V_{f}\right)V_{f}}{\left(k_{f}+G_{r}\right)k_{r}+\left(k_{f}-k_{r}\right)G_{r}V_{f}}\right]\\ \nu_{23}=\frac{2E_{1}k_{T}-E_{1}E_{2}-4\nu_{12}^{2}k_{T}E_{2}}{2E_{1}k_{T}}\\ k_{f}=\frac{E_{f}}{2\left(1-\nu_{f}-2\nu_{f}^{2}\right)}\\ k_{r}=\frac{E_{r}}{2\left(1-\nu_{r}-2{\nu_{r}}^{2}\right)}\\ k_{T}=\frac{\left(k_{f}+G_{r}\right)k_{r}+\left(k_{f}-k_{r}\right)G_{r}V_{f}}{\left(k_{f}+G_{r}\right)-\left(k_{f}-k_{r}\right)V_{f}} \end{array} \end{array}$$

The subscripts *r* and *f* denote matrix and fiber, respectively.

#### 3.3.2. Analytical Prediction of Laminate and Hybrid-Laminate Engineering Elastic Constants

For a laminate, the overall stiffness matrix is expressed as Equation (11).

(11) $$\left[C\right]=\sum_{k=1}^{N}\frac{t_{k}}{t}\left[C_{k}\right]$$

where **[*C_k_*]** and *t_k_* are the stiffness and thickness of the *k*-*th* lamina, respectively; *N* is the number of laminae in the laminate. The compliance matrix **[*S*]** is the inverse of the stiffness matrix **[*C*]**:

(12) $$\left[S\right]={\left[C\right]}^{-1}$$

For a balanced laminate, the overall compliance matrix is:

(13) $$\left[S\right]=\left\{\begin{array}{cccccc} \frac{1}{E_{x}} & \frac{\nu_{yx}}{E_{y}} & \frac{\nu_{zx}}{E_{z}} & 0 & 0 & 0\\ \frac{\nu_{xy}}{E_{x}} & \frac{1}{E_{y}} & \frac{\nu_{zy}}{E_{z}} & 0 & 0 & 0\\ \frac{\nu_{xz}}{E_{x}} & \frac{\nu_{yz}}{E_{y}} & \frac{1}{E_{z}} & 0 & 0 & 0\\ 0 & 0 & 0 & \frac{1}{G_{yz}} & 0 & 0\\ 0 & 0 & 0 & 0 & \frac{1}{G_{xz}} & 0\\ 0 & 0 & 0 & 0 & 0 & \frac{1}{G_{xy}} \end{array}\right\}$$

The equivalent engineering elastic constants of the laminate and hybrid laminate can be computed referring to the method for lamina, i.e.,

(14) $$\begin{array}{c} E_{x}=\frac{1}{S_{11}};E_{y}=\frac{1}{S_{22}};E_{z}=\frac{1}{S_{33}}\\ G_{xy}=\frac{1}{S_{66}};G_{yz}=\frac{1}{S_{44}};G_{xz}=\frac{1}{S_{55}}\\ v_{xy}=\frac{S_{21}}{S_{11}};v_{xz}=\frac{S_{31}}{S_{11}};v_{yz}=S_{32}/S_{22} \end{array}$$

In this study, the method described by Equations (11)–(14) is referred to as the ASPE.

## 4. Results and Discussion

### 4.1. Engineering Elastic Constants of the Lamina

**Figure 5:**
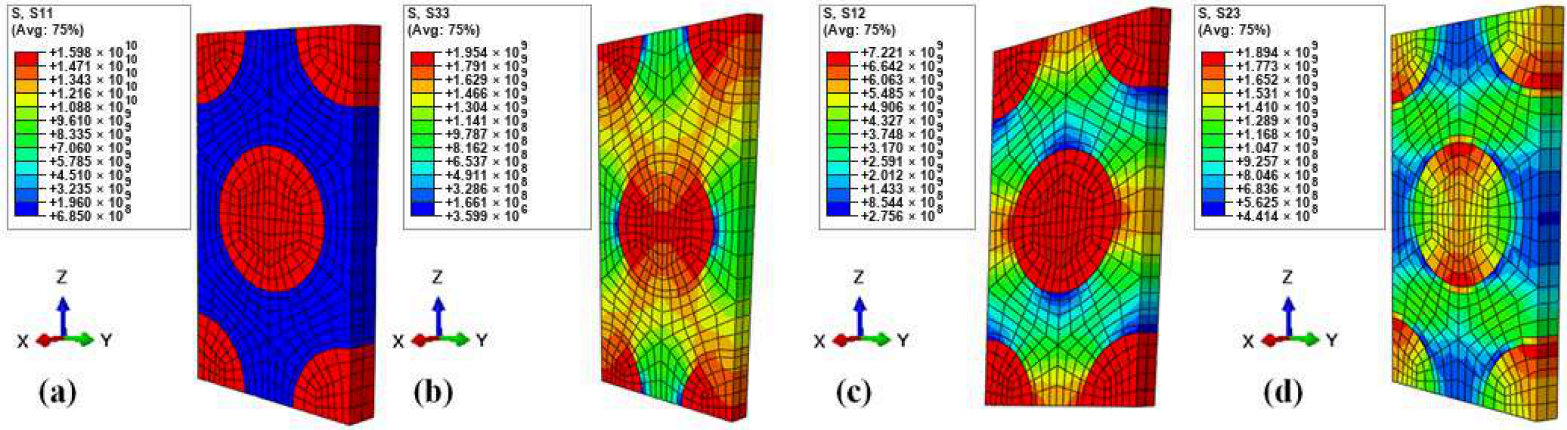
Stress contours of the lamina RVE under different boundary conditions: (**a**) unit normal strain in the longitudinal direction, (**b**) unit normal strain in the transverse direction, (**c**) unit shear strain in the transverse–thickness plane, and (**d**) unit shear strain in the longitudinal–transverse plane.

**Figure 6:**
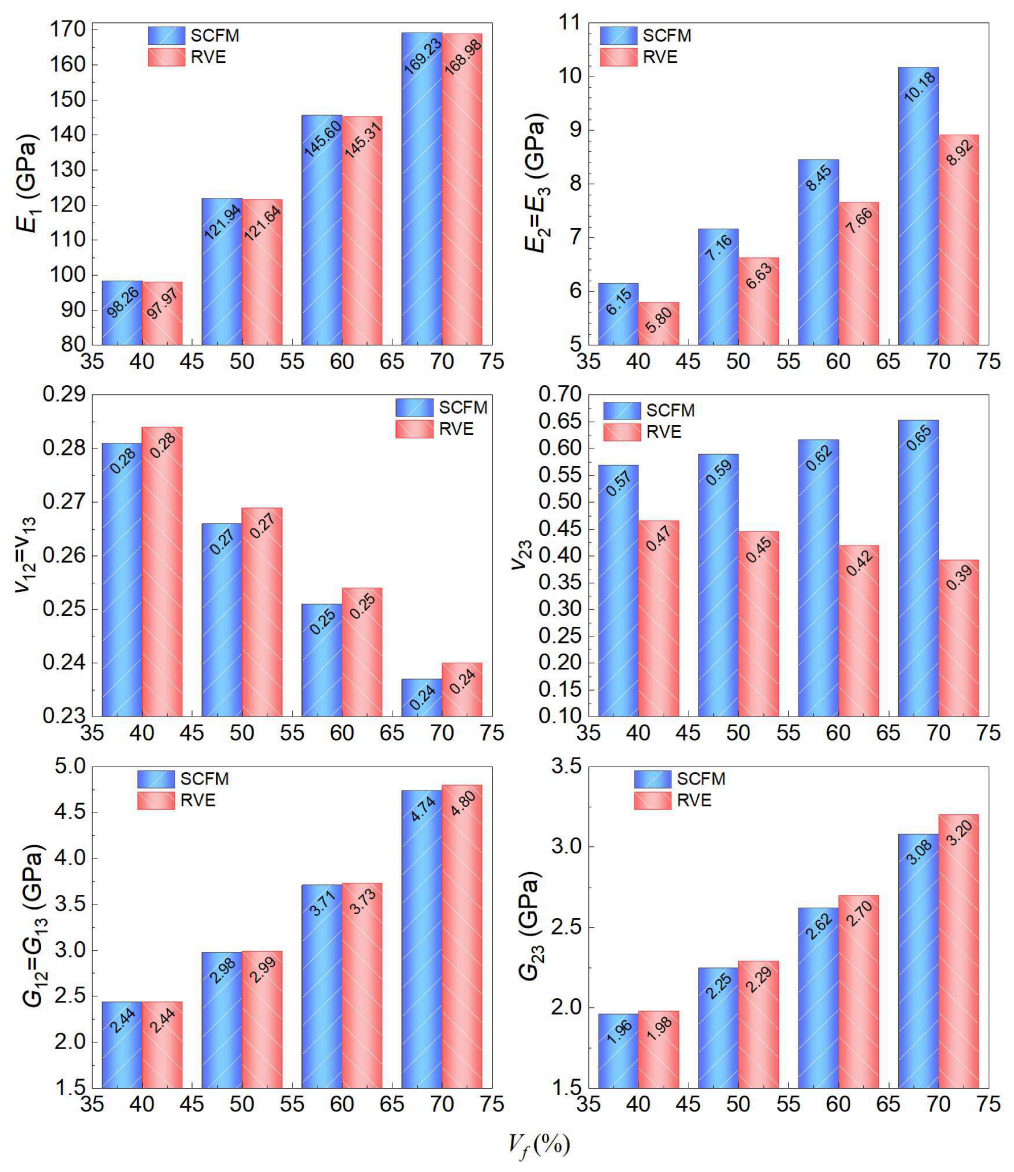
Comparison of lamina engineering elastic constants predicted by SCFM and RVE methods.

Figure 5 shows the stress contours of the hexagonal-packing RVE model of the unidirectional BCFRP lamina under different boundary conditions. The engineering elastic constants of the lamina were extracted by post-processing the simulation results according to Equations (4), (5) and (7). Figure 6 compares the lamina engineering elastic constants estimated by the SCFM analytical model and the FE RVE model as functions of the *V_f_*. Because the unidirectional lamina is transversely isotropic, *E*_2_ = *E*_3_, *G*_12_ = *G*_13_, and *ν*_12_ = *ν*_13_. The two methods yield almost coincident predictions for the fiber-dominated properties *E*_1_, *G*_12_, *ν*_12_ and *G*_23_, whereas noticeable discrepancies are observed for the matrix-dominated transverse modulus *E*_2_ and transverse Poisson’s ratio *ν*_23_. The discrepancy in *ν*_23_ is the most pronounced and grows continuously with *V_f_*. Specifically, the *E*_1_ results from the RVE and SCFM methods are almost identical, with deviations below 2% over the entire *V_f_* range. For *E*_2_, the SCFM model gives higher predictions than the RVE model, and the gap expands as *V_f_* rises. At *V_f_* = 40%, the *E*_2_ values obtained by the RVE and SCFM methods are 6.15 and 6.63 GPa, respectively, giving a difference of 6.03%. At *V_f_* = 70%, the corresponding values increase to 8.92 and 10.18 GPa, and the relative deviation reaches 14.13%. The in-plane Poisson’s ratio *ν*_12_ is also consistently estimated by both approaches. The most significant discrepancy occurs for *ν*_23_: the SCFM prediction climbs from 0.569 at *V_f_* = 40% to 0.653 at *V_f_* = 70%, whereas the RVE prediction falls from 0.47 to 0.39, increasing the relative deviation from 22.10% to 66.16%. For *G*_12_, the two predictions are almost indistinguishable at low *V_f_*, and the RVE model yields only marginally higher values at high *V_f_*, with a maximum deviation of 1.25%. For *G*_23_, the SCFM model consistently gives slightly lower values than the RVE model, with errors of 1.01% at *V_f_* = 40% and 3.75% at *V_f_* = 70%.

The origins of the aforementioned discrepancies can be understood from the load-bearing mechanisms associated with each elastic constant. The *E*_1_ and *ν*_12_ are mainly governed by the axial stiffness of the fibers, which carry most of the longitudinal load (see Figure 5a). Therefore, both methods deliver highly consistent predictions for the longitudinal and in-plane fiber dominated properties. The discrepancy in *E*_2_ arises because the SCFM model fails to capture elastic interactions between adjacent fibers fully. By contrast, the RVE model explicitly resolves the local microstructure and therefore provides a more realistic transverse stiffness prediction (see Figure 5b). For *ν*_23_, the SCFM model estimates the macroscopic response using an equivalent-medium treatment, which cannot capture the strong transverse constraint imposed by densely packed fibers. The RVE model, however, captures the constrained transverse expansion of the matrix at high *V_f_* (see Figure 5c), resulting in a decreasing *ν*_23_ with increasing *V_f_*. This trend is more consistent with the expected constraint effect of densely packed fibers. For *G*_23_, the transverse shear strain in the RVE model must pass through narrow matrix channels between adjacent fibers, producing nonuniform shear deformation and consequently slightly higher macroscopic shear stiffness than that predicted by the SCFM model. For *G*_12_, the load is shared by the fiber and matrix in an in-plane shear dominated mode (see Figure 5d); therefore, the SCFM and RVE predictions remain close.

Overall, the differences between SCFM and RVE predictions arise mainly from the limited ability of the SCFM model to describe transverse fiber constraints and nonuniform matrix deformation. The RVE model incorporates local stress redistribution associated with the actual fiber distribution and therefore gives more reliable predictions for matrix-dominated transverse properties. The SCFM analytical model remains effective for fiber-dominated longitudinal and in-plane shear properties but has limitations in predicting the transverse modulus and transverse Poisson’s ratio. For the fabricated biaxial carbon fiber laminate, *V_f_* was 50.8%, and the corresponding predicted engineering constants of the equivalent unidirectional BCFRP lamina are listed in Table 2.

### 4.2. Engineering Elastic Constants of the Laminate

**Figure 7:**
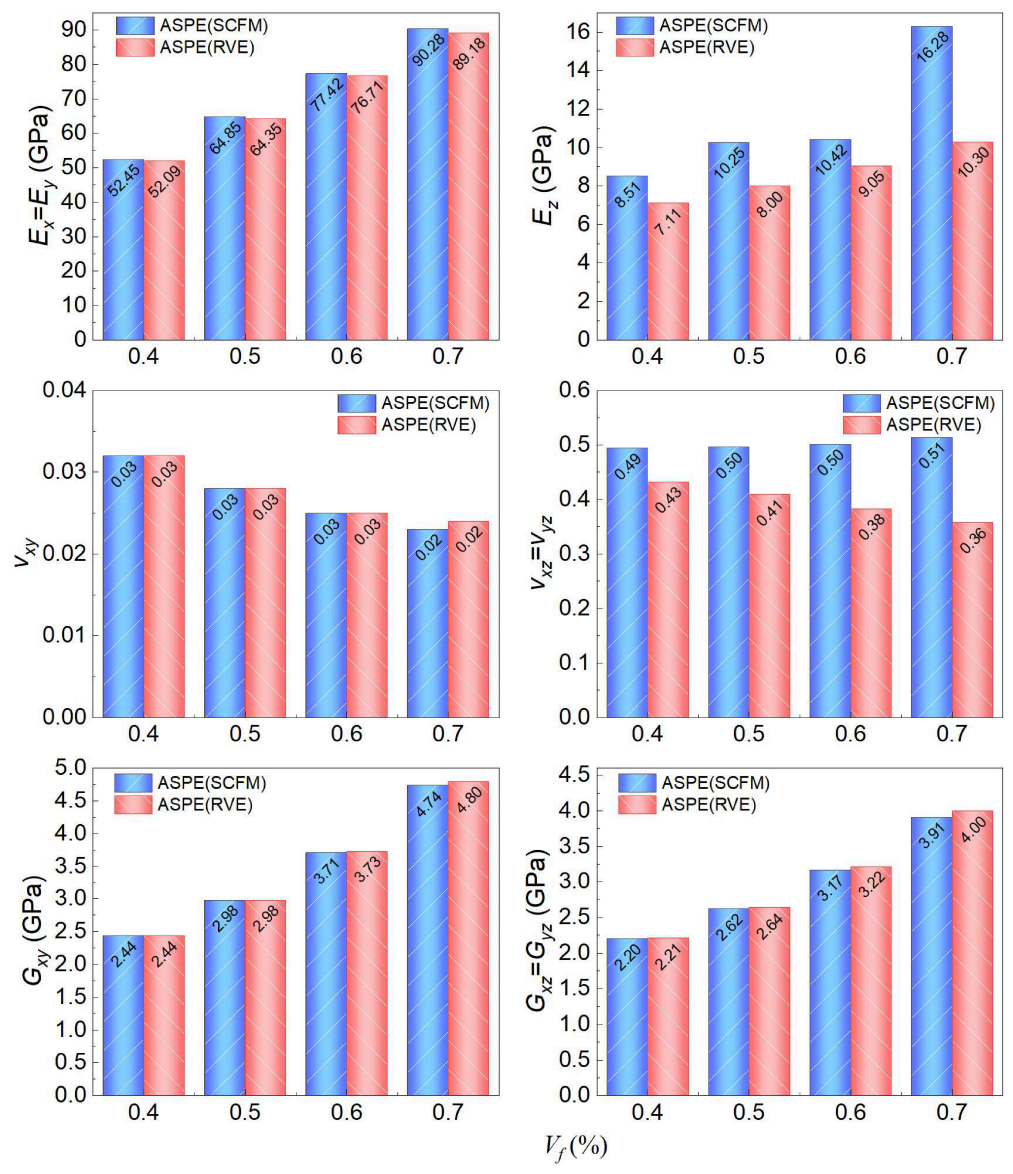
Comparison of equivalent engineering elastic constants of carbon fiber laminates evaluated by ASPE based on SCFM and RVE lamina predictions.

Figure 7 contrasts the equivalent engineering elastic constants of the BCFRP laminate estimated by the ASPE method using lamina properties obtained from the SCFM and RVE methods, hereafter denoted as ASPE(SCFM) and ASPE(RVE), respectively. Because the laminate has a biaxial [0°/90°] layup, the in-plane *x* and *y* directions exhibit identical equivalent properties; therefore, *E_x_* = *E_y_*, *ν_xz_* = *ν_yz_*, and *G_xz_* = *G_yz_*. As shown in Figure 7, ASPE(SCFM) and ASPE(RVE) give very similar predictions for *E_x_* and *G_xy_*, with a negligible difference. The in-plane modulus *E_x_* is mainly governed by the longitudinal lamina modulus *E*_1_, for which the difference between the SCFM and RVE predictions is less than 0.1%, as illustrated in Figure 6. Although the two methods give noticeably different transverse moduli *E*_2_, the contribution of *E*_2_ to the in-plane laminate modulus is limited. The in-plane shear modulus *G_xy_* is primarily determined by the lamina shear modulus *G*_12_; therefore, the difference between ASPE(SCFM) and ASPE(RVE) remains small.

According to classical laminate theory, the through-thickness modulus *E_z_* of a laminate composed of identical laminae is closely related to the lamina through-thickness modulus *E*_3_. However, Figure 7 reveals that *E_z_* predicted by ASPE(SCFM) and ASPE(RVE) is higher than the corresponding *E*_3_ values in Figure 6. Moreover, ASPE(SCFM) produces larger *E_z_* values than ASPE(RVE), and the discrepancy increases with *V_f_*. Specifically, at *V_f_* = 40%, ASPE(SCFM) and ASPE(RVE) predict *E_z_* values of 8.51 GPa and 7.11 GPa, respectively, representing a relative difference of 19.69%; at *V_f_* = 70%, the corresponding values rise to 16.28 GPa and 10.30 GPa, and the relative difference reaches 58.06%. As inferred from Figure 6, this discrepancy mainly originates from the higher *E*_2_ and *G*_23_ values predicted by the SCFM method at the lamina scale. These deviations are directly propagated to the laminate-level *E*_z_ through the [0°/90°] layup.

In addition to *E_z_*, the two ASPE-based predictions also exhibit marked differences in *ν_xz_*. ASPE(SCFM) yields higher *ν_xz_* values than ASPE(RVE), and the gap widens with *V_f_*. At *V_f_* = 40%, the estimated *ν_xz_* values are 0.49 and 0.43, respectively, corresponding to a relative difference of approximately 14.35%. At *V_f_* = 70%, the corresponding values are 0.51 and 0.36, and the relative difference increases to 43.98%. For a biaxial layup, *ν_xz_* depends on both *ν*_12_ and *ν*_23_ of the lamina. As discussed above, the two methods produce nearly identical *ν*_12_ results; thus, the difference in *ν*_xz_ mainly stems from the discrepancy in *ν*_23_. Because the SCFM method predicts higher *ν*_23_ values that increase with *V_f_*, this trend is carried over to the laminate-level *ν_xz_*. For *ν_xy_*, ASPE(SCFM) gives slightly lower values than ASPE(RVE), but the absolute difference remains small. The in-plane Poisson’s ratio *ν_xy_* is dictated by *E*_1_, *E*_2_, and *ν*_12_. Because the *ν*_12_ values from SCFM and RVE are nearly identical, the slight difference in *ν_xy_* mainly arises from the 6–14% difference in *E*_2_. Consequently, ASPE(SCFM) gives slightly lower *ν_xy_* values than ASPE(RVE). Because *ν_xy_* itself is very small, even a minor absolute difference can lead to a relatively large relative error. For *G_xz_*, ASPE(RVE) yields slightly higher predictions than ASPE(SCFM), but the discrepancy remains negligible. At *V_f_* = 40%, the predicted *G_xz_* values are 2.21 and 2.20 GPa, respectively, corresponding to a relative difference of approximately 0.45%. At *V_f_* = 70%, the values are 4.00 and 3.91 GPa, respectively, giving a relative difference of 2.25%. *G_xz_* is strongly affected by the lamina transverse shear modulus *G*_23_. Because the *G*_23_ estimates obtained from the SCFM and RVE methods are close, the difference between the *G_xz_* predictions from ASPE(SCFM) and ASPE(RVE) is also limited.

**Figure 8:**
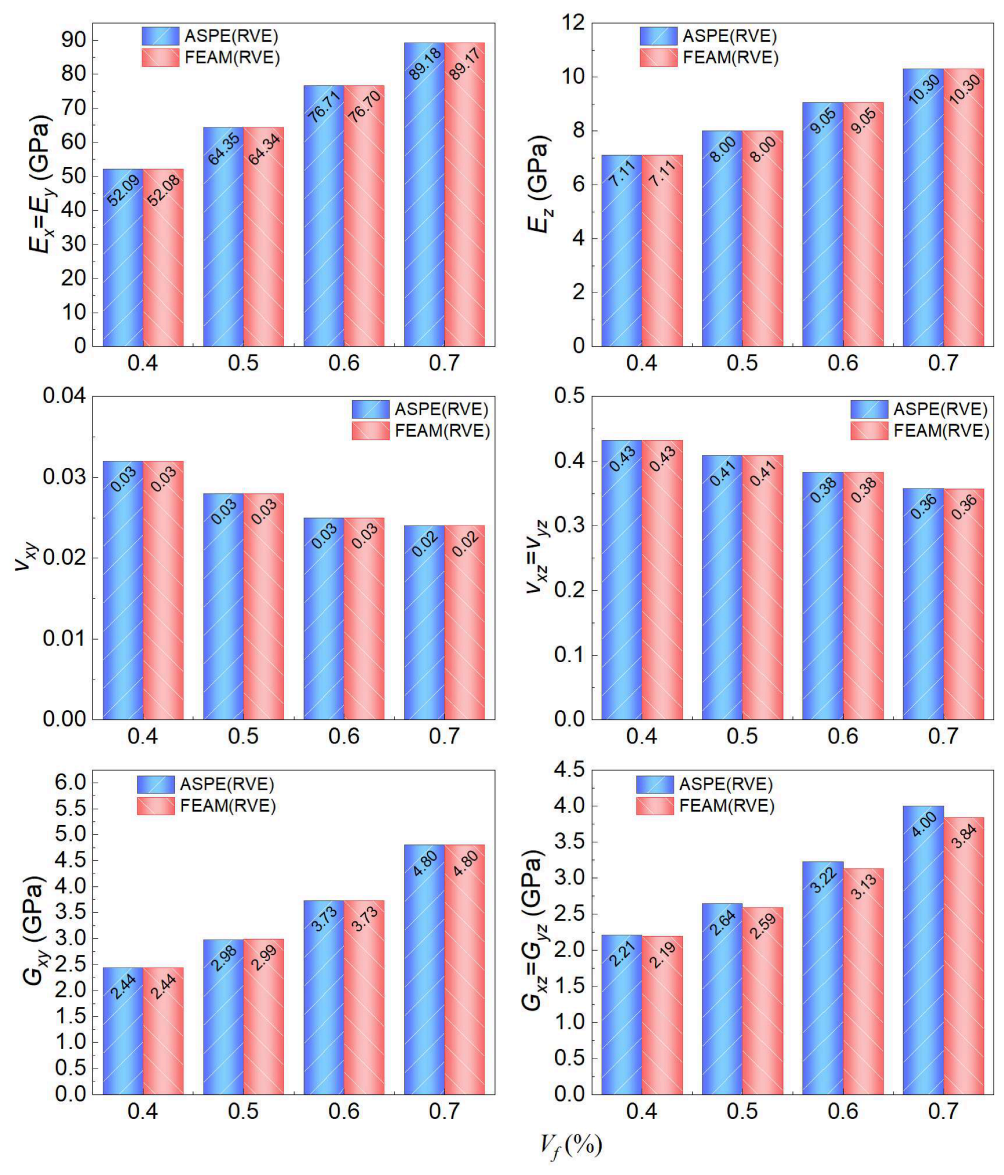
Comparison of equivalent engineering elastic constants of carbon fiber laminates evaluated by ASPE and FEAM based on RVE lamina predictions.

Figure 8 further compares the equivalent engineering elastic constants of the BCFRP laminate predicted by ASPE and FEAM using RVE-derived lamina properties as inputs, hereafter designated as ASPE(RVE) and FEAM(RVE), respectively. For *E_x_*, *E_z_*, *G_xy_*, and *G_xz_*, the differences between the two methods are less than 5%, demonstrating good agreement in modulus prediction. The absolute differences in *ν_xy_* and *ν_xz_* are also small, with negligible differences. Although the small baseline values amplify the relative differences, the *ν_xy_* and *ν_xz_* estimates from the two methods remain close from an engineering perspective. Because ASPE(RVE) and FEAM(RVE) use the same lamina-level input parameters, their differences mainly arise from the calculation strategy. The high consistency between the modulus predictions suggests that ASPE and FEAM have nearly equivalent capability in predicting the equivalent moduli of biaxial laminates when the same lamina properties are used. For Poisson’s ratios, the slight differences can be ascribed to local interlaminar effects and coupled deformation modes captured by FEAM but simplified in the analytical homogenization assumptions.

Table 3 lists the predicted engineering elastic constants of the BCFRP laminate with *V_f_* = 50.8% using ASPE(RVE) and FEAM(RVE), together with the experimentally measured values. For the tensile test, six acceptable data points were recorded. For the shear test, only five acceptable data points remained. It can be seen that *E_x_* values predicted by ASPE(RVE) and FEAM(RVE) are 65.29 GPa and 65.32 GPa, respectively, which differ from the measured average (63.1 GPa), maximum (64.0 GPa), and minimum (62.5 GPa) by approximately 3.5%, 2.0%, and 4.5%. This implies that both methods provide reasonable predictions for the fiber dominated in-plane stiffness. For *G_xy_*, ASPE(RVE) and FEAM(RVE) give 3.03 GPa and 3.04 GPa, respectively, both of which are close to the measured average value of 3.07 GPa. The measured maximum and minimum values are 3.25 GPa and 2.92 GPa, and the predicted values fall well within this experimental range. For *ν_xy_*, the two methods yield 0.027 and 0.028, respectively, which are slightly lower than the measured average value of 0.03 and are closer to the minimum value of 0.029. For *ν_xz_*, *E_z_*, and *G_xz_*, no measured data are available in Table 3; nevertheless, the differences between ASPE(RVE) and FEAM(RVE) remain small, further confirming the consistency between the analytical and numerical homogenization approaches. From this comparison, it can be concluded that both ASPE(RVE) and FEAM(RVE) provide favorable predictions for the in-plane engineering elastic constants. The out-of-plane elastic constants, however, remain unvalidated due to the lack of experimental data, and their experimental verification will be the subject of future work.

### 4.3. Engineering Elastic Constants of Hybrid Laminates

**Figure 9:**
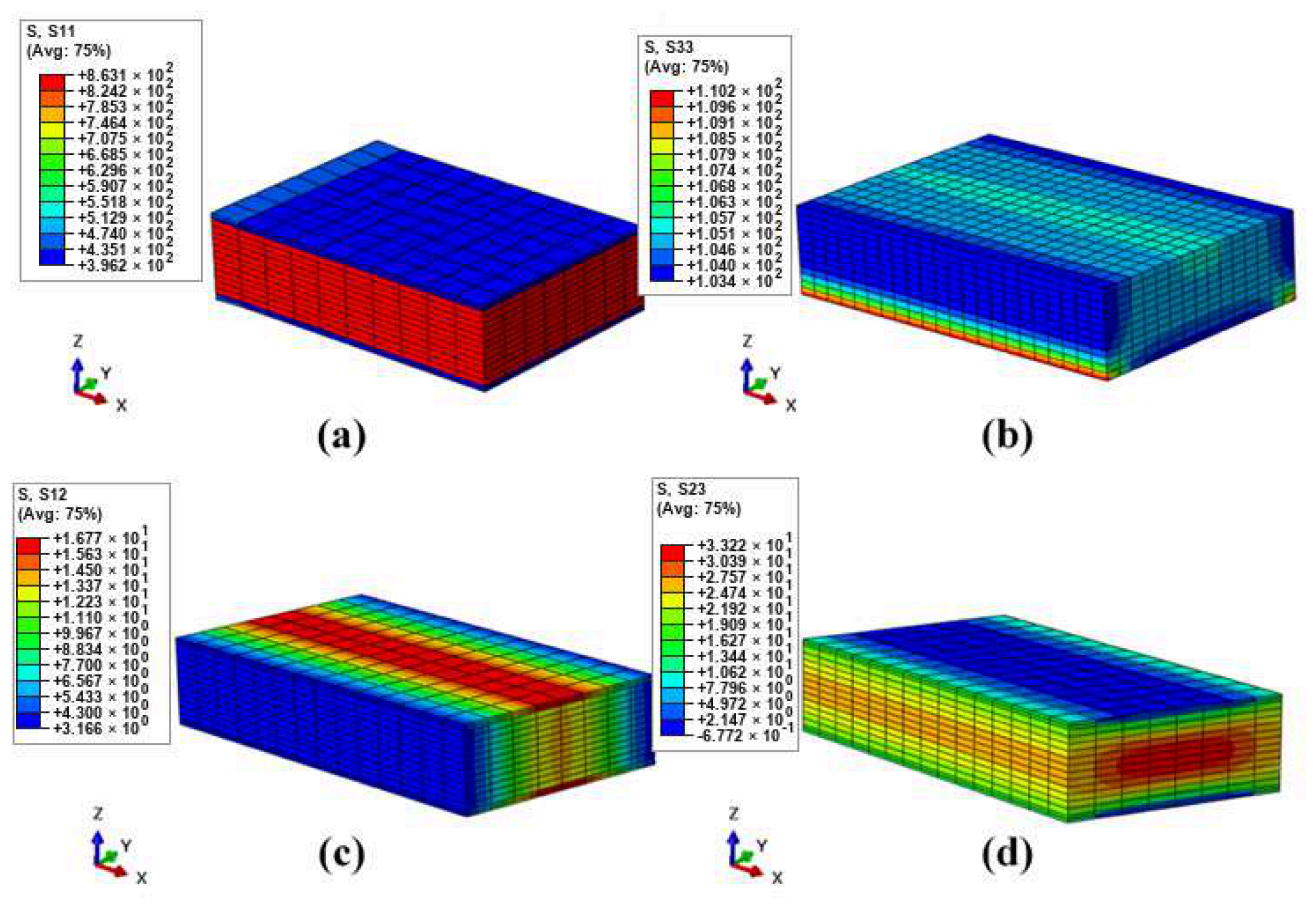
Stress contours of the hybrid-composite RVE under different boundary conditions: (**a**) unit normal strain in the longitudinal direction, (**b**) unit normal strain in the transverse direction, (**c**) unit shear strain in the transverse–through-thickness plane, and (**d**) unit shear strain in the longitudinal–transverse plane.

The actual fiber volume fractions of the GFRP skin and the BCFRP core in the hybrid laminate are 43.0% and 50.8%, respectively. To investigate the effect of fiber volume fractions on the engineering elastic constants, different combinations of fiber volume fractions for the GFRP skin and BCFRP core were considered. In the following analysis, the skin and core are assumed to have the same fiber volume fraction, which varies from 40% to 70%. To predict the equivalent engineering elastic constants of the hybrid laminate, the properties of the GFRP constituents are required. Following the approach in Ref. [30], the equivalent elastic constants of the satin-weave fabric are approximated using a cross-ply model, in which each fabric layer is represented by two unidirectional plies oriented in the warp and weft directions. The properties of the glass fiber and resin are listed in Table 1. The engineering elastic constants of the GFRP lamina are first predicted using the RVE model and then fed into FEAM(RVE) to determine the GFRP skin constants. Table 4 lists the equivalent engineering elastic constants of the GFRP skin laminates at different fiber volume fractions. These GFRP skin properties, together with the FEAM-estimated engineering constants of the BCFRP core, were then used as inputs to calculate the equivalent constants of the hybrid laminate via both the ASPE and FEAM approaches. Figure 9 displays the stress contours of the hybrid-laminate RVE under different boundary conditions. The FE results were post-processed according to Equations (4), (5) and (7) to extract the equivalent engineering elastic constants estimated by the FEAM method.

**Figure 10:**
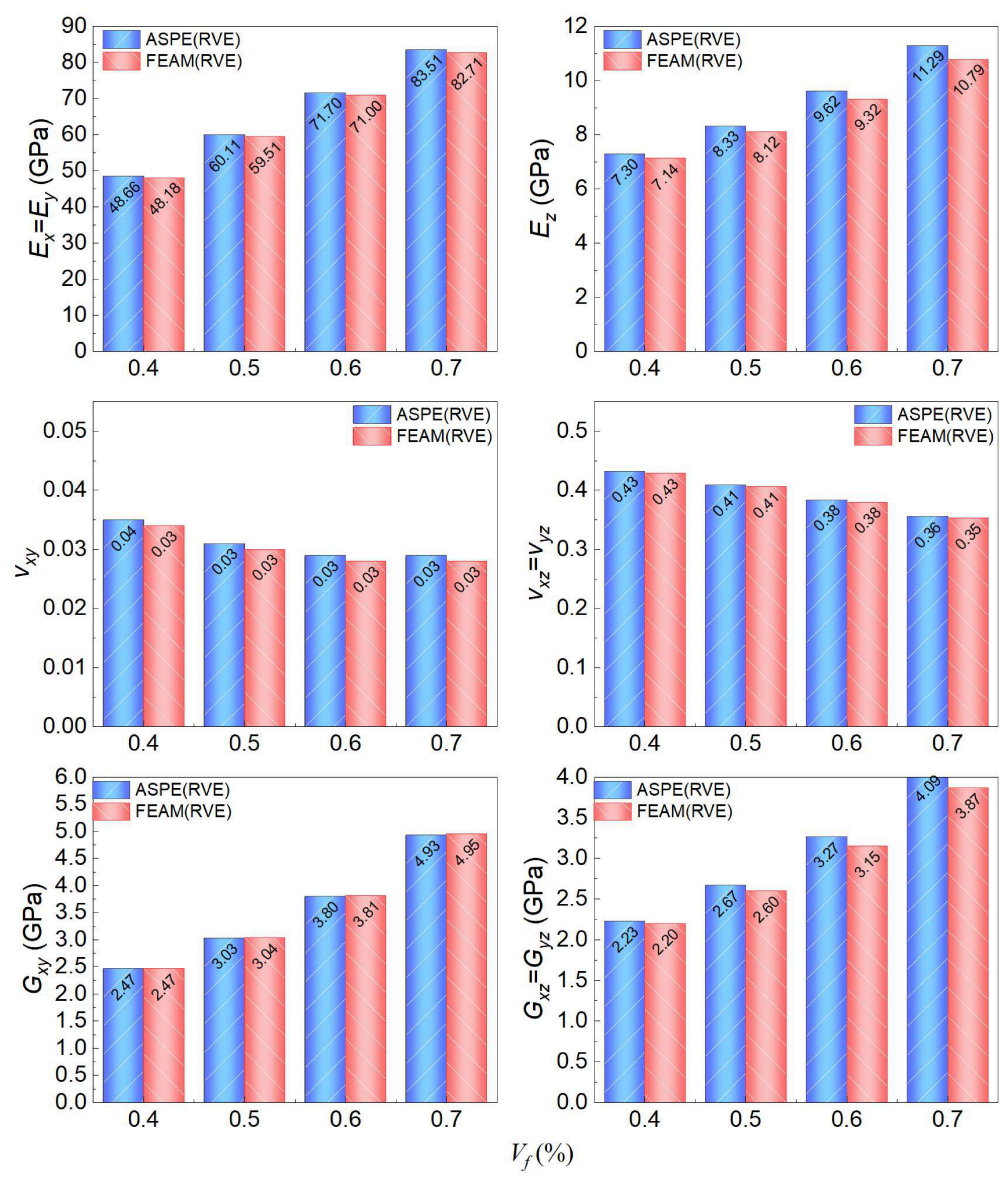
Comparison of equivalent engineering elastic constants of hybrid laminates predicted by ASPE and FEAM using RVE-derived lamina properties.

Figure 10 compares the equivalent engineering constants of the hybrid laminate obtained from ASPE and FEAM. ASPE and FEAM show very small differences in *E_x_* and *G_xy_*, and also for *E_z_* and *G_xz_* at low to medium GFRP skin fiber volume fractions. At higher GFRP skin fiber volume fractions, slight deviations appear in the out-of-plane properties *ν_xz_*, *G_xz_*, and *E_z_*, but the overall differences remain limited. For the in-plane Poisson’s ratio *ν_xy_*, the relative difference appears relatively large because of its very small baseline value; nevertheless, the absolute difference remains acceptable from an engineering perspective. For *E_x_* and *G_xy_*, the in-plane stiffness of the hybrid laminate is mainly dictated by the fiber-dominated longitudinal modulus *E*_1_ of the constituent plies. Because the iso-strain assumption adopted in ASPE is well suited to tension-dominated deformation modes, the ASPE predictions are almost identical to the FEAM results for the in-plane stiffness components. For the out-of-plane constants, the stress and strain fields are more strongly affected by the stiffness mismatch between the GFRP skins and the BCFRP core, as well as by the local interlaminar constraint captured in the FEAM model. This explains why slightly larger deviations appear in *ν_xz_*, *G_xz_*, and *E_z_* at higher GFRP skin fiber volume fractions. Overall, the close agreement between ASPE and FEAM demonstrates that the proposed cross-scale prediction framework can efficiently estimate the equivalent engineering elastic constants of carbon/glass hybrid laminates while maintaining good consistency with finite element homogenization. Table 5 presents the predicted equivalent elastic constants for the manufactured hybrid laminate, which comprises a CFRP core with *V_f_* = 50.8% and GFRP skins with *V_f_* = 43%, obtained using ASPE(RVE) and FEAM(RVE). The differences between the two sets of predictions are minimal, further confirming the reliability of the analytical approach. A direct comparison between experimental measurements and simulation results will be undertaken in future work.

## 5. Conclusions

This study develops a cross-scale homogenization framework for predicting equivalent engineering elastic constants from fiber/matrix constituents to hybrid laminates. The framework combines micromechanics-based analytical models with RVE finite element homogenization and enables progressive property transfer across lamina, laminate, and hybrid-laminate scales. The main conclusions are as follows:

(1) At the lamina scale, the SCFM analytical model overestimates the transverse modulus *E*_2_ and transverse Poisson’s ratio *ν*_23_, and the deviation increases with increasing fiber volume fraction. In contrast, the hexagonal-packing RVE model explicitly resolves local fiber–matrix interactions and provides more reliable predictions for matrix-dominated transverse properties.
(2) At the laminate scale, ASPE(SCFM) and ASPE(RVE) provide very close predictions for in-plane moduli, whereas the out-of-plane properties are sensitive to the transverse lamina inputs. Because the SCFM model overestimates *E*_2_ and *G*_23_ at the lamina scale, these deviations are transferred to the laminate-level out-of-plane properties. Experimental validation confirms that the predicted in-plane tensile modulus, in-plane shear modulus, and in-plane Poisson’s ratio are in good agreement with the measured values.
(3) For hybrid laminates, ASPE(RVE) and FEAM(RVE) show minor differences in predicting the in-plane moduli, out-of-plane moduli, and Poisson’s ratios over the investigated fiber volume fraction range. Slight deviations appear in *ν_xz_*, *G_xz_*, and *E_z_* at high fiber contents, but the overall differences remain small from an engineering perspective.

## Figures and Tables

**Table 1:** Material properties of the fiber and resin.

Properties	Unit	Resin	Carbon Fiber	Glass Fiber
*ρ*	g/cm^3^	1.14	1.81	2.54
*E*_1*f*_ or *E*_1*r*_	GPa	3.24	240	93.5
*E*_2*f*_ or *E*_2*r*_	GPa	3.24	15	93.5
*E*_3*f*_ or *E*_1*r*_	GPa	3.24	15	93.5
*v*_12*f*_ or *v*_12*r*_	-	0.35	0.2	0.22
*v*_13*f*_ or *v*_13*r*_	-	0.35	0.2	0.22
*v*_23*f*_ or *v*_23*r*_	-	0.35	0.3	0.22
*G*_12*f*_ or *G*_12*r*_	GPa	1.2	15	38.32
*G*_13*f*_ or *G*_13*r*_	GPa	1.2	15	38.32
*G*_23*f*_ or *G*_23*r*_	GPa	1.2	5.77	38.32

Note: *E*, *ν*, and *G* denote modulus, Poisson’s ratio, and shear modulus, respectively. Subscripts 1, 2, and 3 indicate the fiber direction, transverse direction, and through-thickness direction, respectively. Subscripts *f* and *r* refer to fiber and resin, respectively.

**Table 2:** Predicted engineering constants of the equivalent unidirectional BCFRP lamina at *V_f_* = 50.8% using the SCFM and RVE methods.

Properties	Unit	SCFM	RVE
*E*_1_	GPa	123.84	123.54
*E*_2_ = *E*_3_	GPa	7.25	6.70
*ν*_12_ = *ν*_13_	—	0.264	0.268
*ν*_23_	—	0.591	0.443
*G*_12_ = *G*_13_	GPa	3.03	3.04
*G*_23_	GPa	2.28	2.32

**Table 3:** Comparison of predicted and measured engineering elastic constants of the BCFRP laminate with *V_f_* = 50.8%.

Properties	Unit	ASPE (RVE)	FEAM (RVE)	Measured (Average, Maximum, Minimum)	Standard Deviation
*E_x_* = *E*_y_	GPa	65.29	65.32	63.1 GPa, 64.0 GPa, 62.5 GPa	0.533
*E_z_*	GPa	8.02	8.07	/	/
*ν_xy_*	—	0.027	0.028	0.030, 0.032, 0.029	0.0013
*ν_xz_* = *ν_yz_*	—	0.412	0.407	/	/
*G_xy_*		3.03	3.04	3.07 GPa, 3.25 GPa, 2.92 GPa	0.138
*G_xz_* = *G_yz_*	GPa	2.65	2.63	/	/

**Table 4:** Equivalent engineering elastic constants of the GFRP skin laminates at different fiber volume fractions.

*Vf*	*Ex* = *Ey*/GPa	*Ez*/GPa	*νxy*	*νxz* = *vyz*	*Gxy*/GPa	*Gxz* = *Gyz*/GPa
40%	23.44	8.25	0.089	0.416	2.65	2.55
43%	25.04	8.77	0.088	0.411	2.83	2.73
50%	28.9	10.21	0.087	0.395	3.34	3.23
60%	34.84	13.08	0.091	0.368	4.33	4.26
70%	41.66	17.62	0.1	0.334	5.92	5.96

**Table 5:** Equivalent engineering elastic constants of hybrid laminate predicted by ASPE(RVE) and FEAM(RVE) (*V_f_* = 50.8% for CFRP core, *V_f_* = 43% for GFRP skin).

Properties	Unit	ASPE (RVE)	FEAM (RVE)
*E*_x_ = *E*_y_	GPa	60.51	59.83
*Ez*	GPa	8.22	8.00
*ν_xy_*	—	0.031	0.03
*ν_xz_* = *ν_yz_*	—	0.410	0.407
*G_xy_*	GPa	3.03	3.01
*G_xz_* = *G_yz_*	GPa	2.67	2.63

## Data Availability

The original contributions presented in this study are included in the article. Further inquiries can be directed to the corresponding author.

## Abbreviations

The following abbreviations are used in this manuscript:

ASPE	Analytical Solution for Predicting Elastic constants
BCFRP	Biaxial Carbon Fiber Fabric Reinforced Polymer composite
FE	Finite Element
FEAM	Finite-Element-Analysis-based Macromechanics
RVE	Representative Volume Element
SCFM	Self-Consistent Field Micromechanics
